# Exploring Hospitals’ Use of Facebook: Thematic Analysis

**DOI:** 10.2196/jmir.9549

**Published:** 2018-05-16

**Authors:** Nima Kordzadeh, Diana K Young

**Affiliations:** ^1^ Robert A Foisie School of Business Worcester Polytechnic Institute Worcester, MA United States; ^2^ Department of Finance and Decision Science Trinity University San Antonio, TX United States

**Keywords:** social media, qualitative research, social networking, health care providers

## Abstract

**Background:**

Although health care organizations such as hospitals and clinics have widely embraced social media as a means to educate the community on health topics and increase patient loyalty and satisfaction, little is known about the content these organizations actually share when using social media channels.

**Objective:**

This study aimed to explore the types of content US hospitals post on their Facebook pages and how hospitals’ Facebook activities differ with regard to content types.

**Methods:**

We collected and thematically analyzed more than 1700 Facebook posts made over a 3-month period by 17 US hospitals. During the first phase, the 2 researchers coded a set of 159 posts and created an initial thematic web of content. During the second phase, the researchers coded the remaining posts and then revised, refined, and validated the initial web of content accordingly. Coding consensus was achieved on 1184 of the 1548 analyzable sampled posts (76.49%).

**Results:**

We identified a list of 13 unique health social media post themes and classified those themes into 3 thematic groups that included *announcing*, *sharing*, and *recognizing* activities. The most frequently used theme was *sharing health information*, which appeared in 35.81% (424/1184) of the posts analyzed. Such posts sought to provide health tips and advice to community members. *Recognizing special days* and *recognizing employees* were the second and third most frequently used themes, respectively, with 14.95% (177/1184) and 11.82% (140/1184) of the posts containing those themes. The frequency of these themes was surprising as the content was geared more toward stakeholders internal to the organization, although most previous literature has focused on social media as a tool to connect with external stakeholders. In addition, we found many of the posts involved more than one theme, and selected sets of themes co-occurred frequently. For example, 25.4% (45/177) of the posts *recognizing special days* also included content to *share health information,* and approximately 38% (32/85) of the posts *announcing research activities* also included content to *share health information*. Finally, we found similarities and differences between the sampled hospitals in terms of the types of content they posted more frequently on their Facebook pages.

**Conclusions:**

Hospitals use Facebook as an inexpensive way to educate people on health and wellness topics and to communicate different types of information and news to the public audience. Hospitals and clinics that are expanding their social media activities or are starting to embark on social media strategies can use the results of this study to better formulate their activities on Facebook.

## Introduction

Over the past decade, social media has infiltrated the health care industry [1]. Hospitals, clinics, and other health care providers use social media channels such as Facebook and Twitter to communicate with their patients and the surrounding community. The main goals of using social media platforms by health care providers such as hospitals and clinics include disseminating health information, tips, and advice; promoting health care services and products; managing brand recognition and reputation; and strengthening ties with their audience [2-5]. Some of these goals such as brand management and recognition are common with organizations in other sectors such as travel and tourism, fashion, and restaurants [6,7]. Other goals such as educating the community about health issues, promoting monetary and organ donation, and sharing patient success stories are specific to the health care domain.

As of April 2018, over 1600 US-based hospitals maintained officially sponsored social media accounts [8]. For example, Johns Hopkins Hospital, a world-class institution based in Baltimore, Maryland, has more than 580,000 Facebook followers [9], whereas the organization’s YouTube channel and Twitter page have more than 47,000 subscribers [10] and 500,000 followers [11], respectively. Use of social media by European-based health care organizations has also increased drastically. A mere 10% of 873 European hospitals sampled in 2010 reported having an institutionally sponsored Facebook account [12], although the following year, over 67% of those institutions had such accounts.

Health care institutions use social media platforms for different purposes. In a survey of 36 US hospitals and health systems conducted by Computer Sciences Corporation’s (CSC) Global Institute for Emerging Healthcare Practices in 2012 [5], respondents were asked *for what purpose does your organization use social media* and *what are your organization’s primary objectives in using social media*? The top responses to the first question were to promote wellness and healthy behaviors as well as marketing services or products. The top responses to the second question included to engage patients or consumers, build greater brand recognition, and attract new customers.

Despite the number of studies concerning how health care organizations use social media, little is known about the content of the posts hospitals make on their social media pages. Among the very few studies that have addressed these questions, Richter et al [13] analyzed hospitals’ Facebook fan page posts to determine which of those institutions had postings related to patient education, staff discussion, staff awards, hospital awards, and consumer engagement. The results showed Facebook was primarily used for educational purposes (91%), followed by staff discussion (76%) and staff awards (63%). These categories used did not emerge from the data but were considered a priori knowledge. In addition, Richter et al [13] employed a binary coding schema to show whether or not there was at least one post related to each of the aforementioned categories. Therefore, their findings did not show what proportion of posts was related to each of the categories. For instance, having one post related to hospital awards was treated the same as having 100 posts related to the category. To fill those gaps and to provide more insights into the actual use of social media platforms in the health care domain, we conducted this study. Our main objectives are as follows: (1) understanding the main content themes of the posts made by major US hospitals on Facebook and (2) examining the differences between hospitals in terms of the types of content they post on their Facebook pages.

## Methods

### Overview

We employed a two-phased thematic analysis to identify the types of content health care institutions post on their institutionally sponsored Facebook pages. Thematic analysis is a qualitative method used to identify, analyze, and report on patterns found in text data [14-16]. Use of the method requires “careful reading and rereading of the data” [17] to identify explicit and implicit meaning embedded within the text [18]. Thematic analysis has been widely employed in health care and medical informatics research. Kneafsey et al [19] used the method to analyze health care professionals’ compassion perceptions to reveal 4 overarching components of the concept. Holm and Severinsson [20] used thematic analysis to identify key patterns found in geriatric patients’ narratives regarding their experiences of surviving with depression. Hickey et al [21] employed thematic analysis in their mixed-method, longitudinal study of factors that influence nurses’ career choices and aspirations. Finally, Amann and Rubinelli [22] used thematic analysis to understand the views of community managers on knowledge cocreation in Web-based health communities for people with disabilities.

The thematic analysis process involves 6 steps with iteration allowed between steps [14,23,24]. The process begins with the research team becoming familiar with the data to generate an initial set of themes to code the data. Next, the researchers read and reread the text data to tag individual elements with theme codes. When using the method inductively, tagging can be a highly iterative activity with the researchers actively updating and revising the list of theme codes and data elements throughout the search process [18]. Once all data elements have been tagged, the final list of themes are reviewed and corresponding names and definitions developed. Finally, a graphical thematic web of content is developed to visually depict the relationship between the data and the final list of identified themes. In this study, we performed 2 phases of thematic analysis. In the first phase, we developed an initial thematic web of content using a small sample of Facebook posts. In the second phase, we used a larger sample to validate the thematic web developed in the first phase of the study.

### Sampling and Data Collection

To identify the types of content shared by health care institutions, we first collected the Facebook posts made during a 3-month period (March 2014 to May 2014) by a set of highly ranked, US-based hospitals, selected from the US News Best Hospitals 2013-2014 list. We chose the 3-month time window because a shorter window could raise the possibility of month-specific patterns in the data, which would decrease the generalizability of the results. Conversely, a longer window would result in a much larger dataset, making the content analysis process significantly harder and more complex. It would also potentially increase the likelihood of errors and time to publication of the results.

From the US News Best Hospitals 2013-2014 list, we selected the top 10 institutions from each category. We then eliminated duplicate institutions and removed all institutions offering services in a limited health care domain such as psychiatry. We felt this last step was necessary as we were most interested in how general care hospitals use social media and believed specialty institutions would be more specialty topic focused. This resulted in a final list of 54 general medicine hospitals.

Next, we searched Facebook to determine which of those institutions offered an institutionally sponsored Facebook page. A total of 28 hospitals met that criteria; however, there was wide diversity in the popularity of the institutions’ Facebook main pages. Three institutions, including Cleveland Clinic, Mayo Clinic, and Johns Hopkins Hospital, had very popular main pages with 1,008,069; 517,519; and 205,115 main page likes, respectively. Seventeen institutions had between 5000 and 65,000 main page likes, and the remaining 8 institutions had fewer than 5000 main page likes.

To ensure we compared data from similar organizations, we chose to ignore the 3 institutions with highly popular main pages and the institutions with fewer than 5000 main page likes. We included, instead, the 17 organizations with between 5000 and 65,000 main page likes in our sample frame. We chose these thresholds because fewer than 5000 likes may indicate the organization is not following a systematic social media strategy and hence may not be used as a reliable benchmark for other medical institutions. Moreover, Mount Sinai Medical Center with nearly 65,000 page likes was the most popular page after Mayo Clinic’s main page with over 500,000 likes. Thus, we felt the difference between those two represented a natural cut-off point in the data. Table 1 presents information about the sampled hospitals and the data collected from each hospital’s Facebook page.

### Data Analysis

#### Phase 1

For the first phase of the study, our goal was to identify an initial list of themes that represented the intent or purpose of the posts made by hospitals on their institutionally sponsored Facebook pages. To do so, we randomly selected 5 of the 17 organizations included in our sample frame. The selected institutions included Mount Sinai Medical Center, Rush University Medical Center, Massachusetts General Hospital, Yale-New Haven Hospital, and National Jewish Health. These 5 hospitals made a total of 159 posts between March 1, 2014, and April 30, 2014, on their Facebook pages. Following the thematic analysis steps proposed by Braun and Clarke [14] and widely adopted in the extant literature (eg, [22-24]), we analyzed the 159 posts.

**Table 1:** Sample characteristics.

Hospital’s name^a^	Number of page likes as of the data collection date	Number of posts			
		March 2014	April 2014	May 2014	Total
Mount Sinai Medical Center	64,932	21^b^	22^b^	54	97
Barnes-Jewish Hospital Washington University	62,936	27	43	37	107
New York-Presbyterian University Hospital of Columbia and Cornell	42,159	23	36	47	106
Rush University Medical Center	30,254	16^b^	17^b^	13	46
University of Pittsburgh Medical Center	27,337	39	53	58	150
Stanford Hospital and Clinics	26,316	14	15	14	43
Brigham and Women's Hospital	20,109	36	45	30	111
National Jewish Health, Denver-University of Colorado Hospital	19,074	5^b^	18^b^	54	77
Shepherd Center	15,153	31	35	36	102
Cedars-Sinai Medical Center	12,300	25	25	32	82
Yale-New Haven Hospital	10,540	11^b^	16^b^	28	55
Northwestern Memorial Hospital	9452	47	84	58	189
Thomas Jefferson University Hospital	8326	74	65	73	212
Massachusetts General Hospital	7574	8^b^	25^b^	20	53
NYU Langone Medical Center	7185	26	31	28	85
Florida Hospital	6977	40	34	27	101
Magee-Women’s Hospital of UPMC	5622	36	37	33	106
*Total*	*376,246*	*479*	*601*	*642*	*1722*

^a^The hospitals are ordered in the table based on the number of their followers (ie, page likes) on Facebook.

^b^The numbers are associated with the posts analyzed during the first phase of the study.

**Figure 1:**
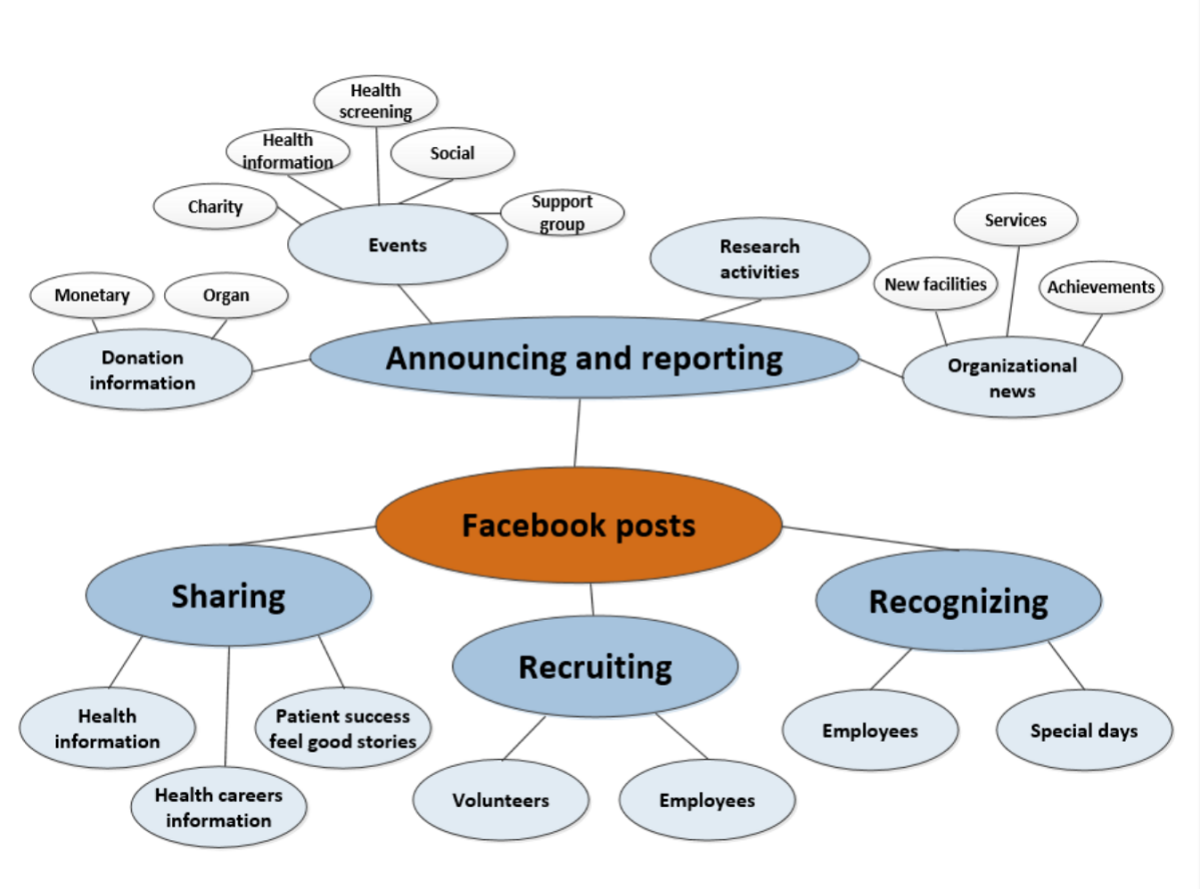
Thematic web of content (phase 1).

We first familiarized ourselves with the sample data and independently generated initial lists of theme codes. We then met, compared our lists, discussed differences, and settled upon an initial set of codes to use when tagging the sampled data elements. In addition, we agreed each researcher could add codes to the list, and we would discuss those additions later. Next, we each independently reviewed each of the sample data elements and inspected any photos or videos included in the post to discern meaning. We then tagged it with up to 3 theme codes believed to be relevant to the post. We met on 2 separate occasions during the coding process to compare our updated lists of theme codes, resolve differences, and discuss the processes we each employed. During the early course of those discussions, we agreed to shift away from applying a strictly semantic lens (word meaning) when analyzing the data elements and toward a more interpretive lens (statement intent) [25]. This was necessary, as intent was not always clearly conveyed in just the post text. For example, the text of one post announced the date and time of an upcoming social event; however, the attached image contained a graphic indicating the event was sponsored by an organization concerned with organ donation. An interpretive lens was necessary to induce meaning from the photo, which ultimately resulted in coding the post as both *announcing a social event* and *promoting organ donation*.

We made several iterations through the entire sample. Once we reached consensus on the final set of codes and coding of all data elements, we developed names and definitions for each theme code. We then spent time analyzing the individual theme codes to identify similarities in purpose and intent to group the codes into theme content categories and subcategories. Finally, we used that information to develop our thematic web of content (Figure 1).

#### Phase 2

According to Lincoln and Guba [26], the validity and soundness of qualitative research is determined based on the credibility, transferability, and confirmability of the results. In the context of thematic analysis, these criteria can be achieved by conducting a thematic content analysis on a second dataset to confirm the findings of the first round of analysis [26]. Thus, to enhance the overall robustness of the phase 1 thematic web of content and to validate the list of content themes identified, we conducted a second thematic analysis on the remaining posts made by the 17 hospitals between March 1, 2014, and May 30, 2014. As such, for the 12 institutions not included in the first phase, we analyzed all posts made during the 3-month sample period. For the 5 institutions included in the first phase of the study, we only analyzed the posts made in May 2014. As such, in the second phase, we analyzed 1563 Facebook posts.

Over the course of several months and using the theme categories and subcategories identified during the first phase of the study, we each independently reviewed and tagged each data element in the new sample with up to 3 theme codes deemed to be most relevant to the post intent. Again, an interpretive approach was applied with each of us reading every post multiple times to induce the underlying purpose before assigning codes. In cases where URL links, photos, or videos were included, we accessed those links and reviewed the multimedia content to ensure any meaning included in those items was considered. As potential new codes were identified during the coding process, we tagged the corresponding elements with the *Other* code and a note specifying a possible code addition.

At 3 points during the phase 2 coding process, we met to discuss our progress, issues we had encountered, and any elements tagged with the *Other* code. During those discussions, we found that no elements were consistently tagged with the *Other* code by both researchers. Accordingly, no additional codes were added to the agreed upon list. Again, we applied a highly iterative process in which individual data elements were reviewed multiple times by each researcher.

## Results

### Phase 1

At the end of the first phase, we had identified 33 unique theme codes. Of the 159 posts, we did not code 6 posts as they were cover photo changes and did not contain any textual content. We tagged most posts (122) with a single theme code, whereas 29 posts were tagged with 2 theme codes and 2 posts were tagged with 3 theme codes. Review, comparison, and grouping of the individual theme codes resulted in a list of 11 theme categories: *donation information, events, research activities, organizational news, recognizing special days, recognizing employees, recruiting employees, recruiting volunteers, sharing patient success stories, sharing health career information,* and *sharing health information*. Moreover, 3 of the 11 categories could be further broken into multiple subcategories. We identified 2 subcategories within the donation information category, 5 subcategories within the event category, and 3 subcategories within the organizational news category. We further organized the 11 theme categories into 4 theme purpose groups, which included *announcing and reporting*, *sharing*, *recognizing*, and *recruiting activities*.

We found that 2 of the individual theme codes (*issue debate* and *nonmedical product promotion*) did not fit within any of the identified theme categories. A single post was coded as *issue debate* and 2 posts were coded as *nonmedical product promotions*. The *issue debate* post was related to the pediatric immunization issue. The *nonmedical product promotion* posts concerned t-shirt sales at a single hospital. As we did not believe either of those codes were of significant importance for our study, we did not include them in the thematic web of content but did retain the codes for use in phase 2 of the study. The *announcing and reporting* theme purpose group involved posts communicating information concerning *donations*, upcoming or past *events*, institution *research activities*, and *organizational news*. A total of 94 of the collected posts (59%) involved *announcing or reporting* activities. Table 2 lists the category and subcategory themes identified within the *announcing and reporting* purpose group and provides sample posts for each of the subcategories and categories identified within that group.

The *sharing* purpose group involved posts disseminating information concerning *health information*, *health careers information*, and *patient success stories*. A total of 44 posts (28%) involved *sharing information*. The *recognizing* purpose group involved posts *acknowledging employees* and *special days*. Furthermore, 42 posts (26%) involved *recognition* activities. Finally, the *recruiting* purpose group involved posts soliciting *new employees and volunteers*. Only 3 posts (2%) involved *recruiting* activities. Table 3 lists the theme categories identified within the *sharing*, *recognizing*, and *recruiting* purpose groups and provides sample posts for each category.

Figure 1 presents the thematic web of content we developed during phase 1 of the study. This model shows the relationships between the identified theme purpose groups, categories, and subcategories.

### Phase 2

We began phase 2 with the 20 theme codes identified during the first phases of the study plus the *Other* code for a total of 21 theme codes. These theme codes represented all subcategories and single-dimensional categories included in the phase 1 thematic web, as well as the *nonmedical product promotion*, and the *issue debate* theme codes that were not included because of low representativeness. During the coding process, we found we were consistently assigning the *organizational news-* *achievements* and the *organizational news-* *new facilities* concurrently. This was because new facilities announcements were made in conjunction with new services that were promoted as institutional achievements. Accordingly, we decided to combine the 2 codes into a single theme code midway through the coding process.

Of the 1563 posts analyzed, 15 posts were not assigned any theme code, as they were cover photo changes and did not contain analyzable content. The first researcher tagged 988 posts with a single theme code, 487 posts with 2 theme codes, and 73 posts with 3 theme codes. The second researcher tagged 1063 posts with a single theme code, 415 posts with 2 theme codes, and 70 posts with 3 theme codes. We achieved coding consensus on 1184 of the sampled posts (76.49%). We calculated interrater reliability based on Cohen kappa statistic using a weighted average of each theme code’s kappa value [27]. The resulting reliability was measured at 73.44%, (κ=.7344), which is considered a very good level of interrater reliability [28].

**Figure 2:**
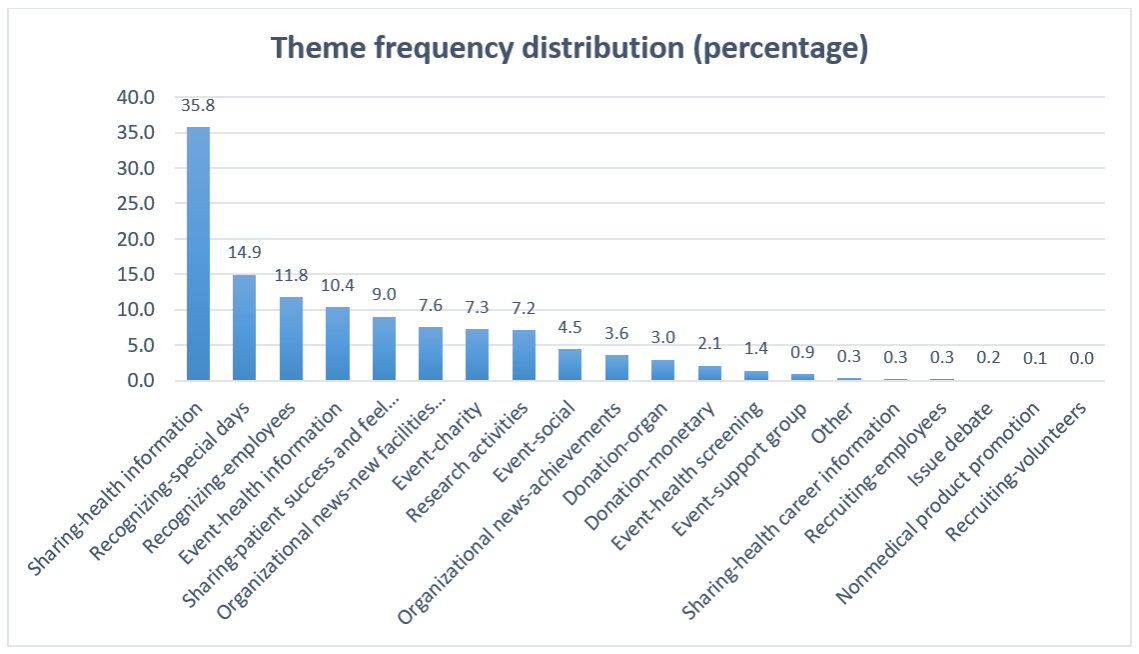
Theme frequency distribution by percentage.

As our goal was to generate a validated set of content theme codes the 2 researchers achieved a consensus upon, we felt it was important to proceed with only the data elements where we both agree on their themes. Accordingly, we eliminated the 337 data elements to which we assigned different codes. This reduced the analyzable sample size to 1184 data elements. Next, we conducted frequency analysis on this reduced sample to determine how representative each of the 20 theme codes was. In line with our phase 1 results, the *sharing-health information* code was assigned most frequently, with 35.81% of the data elements representing this theme code. *Recognizing-special days* and *recognizing-employees* were the second and third most frequently assigned codes, representing 14.95% and 11.82% of the data elements, respectively. This too was in line with our phase 1 results where *recognizing-special days* was also the second most frequently assigned code and *recognizing-employees* was the fifth most frequently assigned code. Figure 2 shows the percent frequency of each of the content theme codes assigned during the phase 2 analysis.

**Table 2:** Announcing and reporting theme categories, subcategories, and example post excerpts.

Category		Count	Example post excerpt
**Donation**			
	Monetary	5	*Thank you New York Rangers’ Brad Richards for your generous gift of US $25,000 to support our Pediatric #Palliative Care Program! *
	Organ	6	*What does it feel like to save a life? At our #OrganDonor Appreciation Ceremony, donors shared personal stories of why they gave the #GiftofLife.*
**Events**			
	Charity	6	*Are you a Tough Mudder? Learn how you can participate in and support the Student Veteran Society from Columbia College’s Paved in Mud campaign to benefit the Center for Veterans and Their Families at Rush.*
	Health information	17	*Arthritis in your knee can make even simple daily tasks hard to complete. Join an orthopedic surgeon and rheumatologist at Rush on April 9 to learn about nonsurgical and surgical treatment options.*
	Health screening	9	*Join our #HealthFair this Saturday from 10 AM* *-3* *PM* *for FREE health screenings including #HepC, #Diabetes, #BloodPressure, and #Cholesterol.*
	Social	16	*National Jewish Health and the LA Professional Services Black and White Ball presented by Debbie & Stu Steinberg and Paul Zaffaroni.*
	Support groups	1	*CPAP Support Group May 10 at 10 AM* *in MDT National Jewish Health in Denver, Colorado.*
Research activities		9	*A research team is investigating whether the body’s own immune system can be encouraged to mount a defense against #cancer before healthy tissue is damaged.*
**Organizational news**			
	New facilities	7	*Thank you to everyone who celebrated the opening of our new #Maternal#Fetal Medicine Center and #Pediatric Specialty Center expansion at One Long Wharf in New Haven last night. *
	Services	8	*Here is the Department of Maternal-Fetal Medicine's Photo of the Month! Our MFM specialists and registered sonographers use state-of-the-art 3D or 4D ultrasounds*
	Achievements	10	*Mass General is proud to again be listed on DiversityInc’s list of Top 10 Hospital Systems. DiversityInc, a publication about diversity and business, ranked Mass General 7th on its annual list of top employers, noting the hospital’s commitment to mentoring minority physicians and nurses. For more information on the DiversityInc list, visit link*

**Table 3:** Sharing, recognizing, and recruiting theme categories and example post excerpts.

Theme purpose and category		Count	Example post excerpt
**Sharing**			
	Health information	31	*How much fiber is on your plate? You need 25-35 g/day. Rethink your #salad*
	Patient success and feel good stories	11	*Amazing story about a 3-year old who beat the odds following a failed kidney transplant.*
	Health career information	2	*What is a Child Life specialist? Learn more: link*
**Recognizing**			
	Employees	28	*YNHH’s Friedlaender earns top leadership honor from American Academy of#Orthopaedic #Surgeons. http://ow.ly/uAGHV*
	Special days	14	*Everyone loves to get a thank you note, including your doctor. You can express your gratitude by sending your doctor at Rush a thank you eCard and making a gift in his or her honor in celebration of National Doctor’s Day, which is Sunday, March 30.*
**Recruiting**			
	Volunteers	2	*National Jewish Health is currently seeking volunteers who are looking for a challenging opportunity to use their retail and/or professional skills in Nan and Dollie’s Gift Shop.*
	Employees	1	*Do you want to become a part of Mount Sinai and help transform the #healthcare landscape? Register for our #Research #OpenHouse in May*

**Figure 3:**
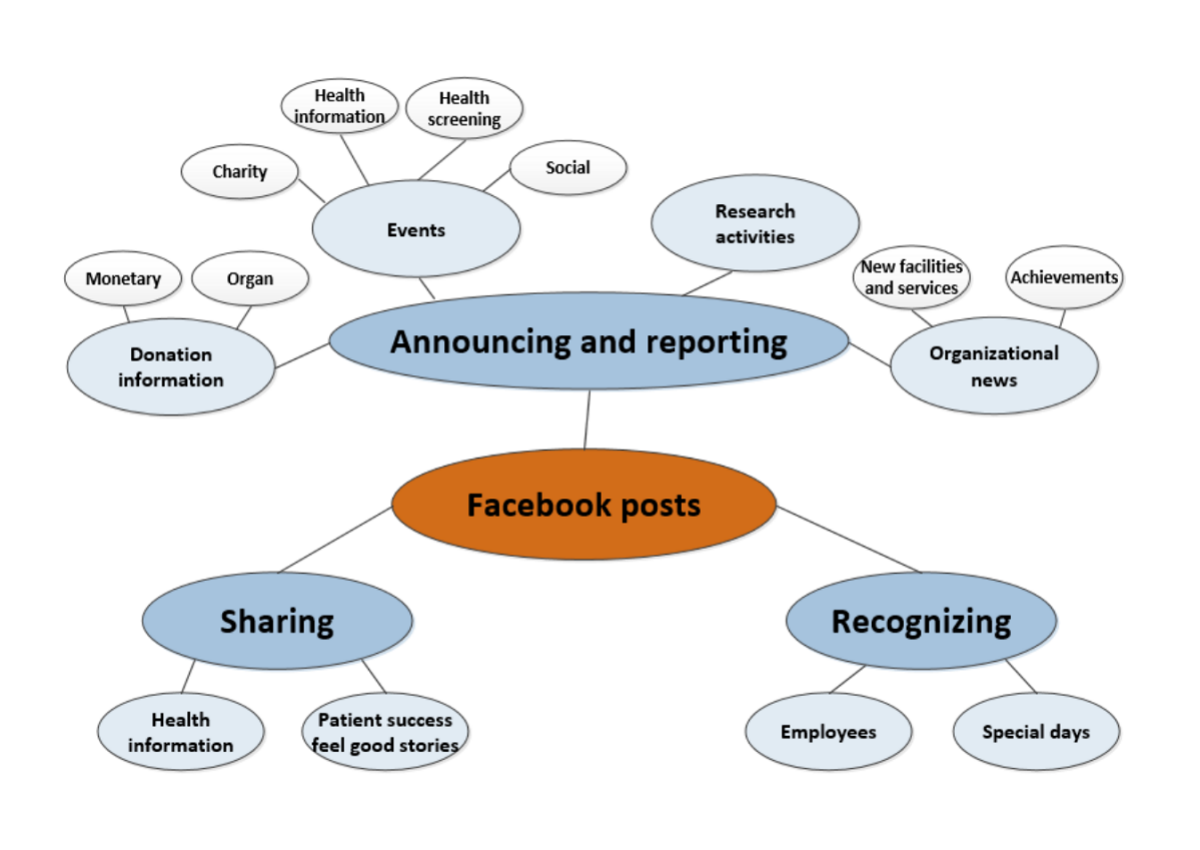
Thematic web of content (phase 2).

As 6 of the identified theme codes each represented less than 1% of the posts in the sample, we decided those codes were not representative of the entire sample and should not be included in our validated thematic web of content. Accordingly, we dropped the *announcing and reporting events-support groups, sharing-health career information, recruiting employees, issue debate, nonmedical product promotion,* and *recruiting volunteers* theme codes. In addition, we agreed to drop the *Other* code as no distinct themes arose from the items tagged with that code. Finally, we updated the thematic web of content to reflect this validated set of theme code categories and subcategories. Figure 3 presents the revised model.

Next, we examined the frequency distribution of the post themes for each hospital and compared the top 3 themes (in terms of frequency of use) that each of the 17 hospitals included in their Facebook posts. The results (Table 4) demonstrated *sharing-health information* was consistently among the top 3 most frequently used themes by the hospitals. The only exception was New York-Presbyterian Hospital that had used the theme in only 6 of the 55 posts; thus, it was not among that hospital’s top 3 themes.

**Table 4:** Most frequently used themes by each hospital.

Hospital^a^	Total number of posts analyzed in phase 2	Theme and n (%) of posts containing the theme		
		First most frequent	Second most frequent	Third most frequent
Mount Sinai Medical Center	38^b^	Sharing health information, 9 (24)	Recognizing employees, 9 (24)	Announcing or reporting event-health information, 6 (16)
Barnes-Jewish Hospital Washington University	68	Sharing health information, 22 (32)	Recognizing special days, 19 (28)	Sharing patient success stories, 16 (24)
New York-Presbyterian University Hospital of Columbia and Cornell	55	Announcing or reporting events-health information, 10 (18)	Recognizing special days, 9 (16)	Sharing patient success stories, 7 (13)
Rush University Medical Center	10^b^	Recognizing special days, 5 (50)	Sharing health information, 4 (40)	Recognizing employees, 3 (30)
University of Pittsburgh Medical Center	111	Sharing health information, 52 (46.9)	Recognizing special days, 20 (18.0)	Announcing or reporting organ donation and recognizing employees, 9 (8.1)
Stanford Hospital and Clinics	35	Sharing health information, 12 (34)	Recognizing special days, 10 (29)	Sharing patient success stories, 3 (9)
Brigham and Women’s Hospital	92	Sharing health information, 38 (41)	Announcing or reporting events-charity, 11 (12)	Announcing or reporting research activities, 11 (12)
National Jewish Health, Denver-University of Colorado Hospital	54^b^	Sharing health information, 22 (41)	Announcing or reporting research activities, 9 (17)	Recognizing employees, 4 (7)
Shepherd Center	84	Sharing patient success stories, 23 (27)	Sharing health information, 17 (20)	Announcing or reporting event-charity, 14 (17)
Cedars-Sinai Medical Center	68	Sharing health information, 37 (54)	Announcing or reporting research activities, 8 (12)	Announcing or reporting events-health information, 7 (10)
Yale-New Haven Hospital	27^b^	Sharing health information, 10 (37)	Recognizing special days, 6 (22)	Recognizing employees, 5 (19)
Northwestern Memorial Hospital	139	Sharing health information, 42 (30.2)	Recognizing special days, 21 (15.1)	Sharing patient success stories, 17 (12.2)
Thomas Jefferson University Hospital	175	Sharing health information, 68 (51.1)	Recognizing employees, 38 (21.7)	Recognizing special days, 26 (14.9)
Massachusetts General Hospital	12^b^	Sharing health information, 6 (50)	Announcing or reporting events-charity, 2 (17)	Recognizing special days, 1 (8)
NYU Langone Medical Center	68	Sharing health information, 21 (30)	Recognizing employees, 11 (16)	Announcing or reporting research activities, 11 (16)
Florida Hospital	70	Sharing health information, 24 (34)	Announcing or reporting events-health information, 18 (26)	Recognizing special days, 11 (16)
Magee-Women’s Hospital of UPMC	78	Sharing health information, 34 (44)	Recognizing special days, 22 (28)	Recognizing employees, 15 (19)

^a^The hospitals are ordered in the table based on the number of their followers (ie, page likes) on Facebook.

^b^The numbers are associated with the posts made only in May 2014 because the March and April posts of those hospitals were analyzed during the first phase of the study.

**Table 5:** Theme co-occurrence rates. N=number of posts tagged with the theme. N/A: not applicable.

Theme^a^	Sharing Health Information, n (%)	Recognizing Special Days, n (%)	Recognizing Employees, n (%)	Event Charity, n (%)	Event Social, n (%)
Sharing Health Information (N=424)	N/A	45 (10.6)	8 (1.9)	0 (0.0)	0 (0.0)
Recognizing Special Days (N=177)	45 (25.4)	N/A	21 (11.9)	1 (0.6)	4 (2.3)
Recognizing Employees (N=140)	8 (5.7)	21 (15.0)	N/A	1 (0.7)	1 (0.7)
Organizational News New Facilities and Services (N=90)	11 (12)	3 (3)	6 (7)	0 (0)	0 (0)
Research Activities (N=85)	32 (38)	2 (2)	5 (6)	1 (1)	0 (0)
Event Charity (N=86)	0 (0)	1 (1)	1 (1)	N/A	10 (12)
Event Social (N=53)	0 (0)	4 (8)	1 (2)	10 (19)	N/A
Organizational News Achievements (N=43)	2 (5)	2 (5)	8 (19)	0 (0)	0 (0)
Donation Organ (N=35)	3 (9)	4 (11)	1 (3)	4 (11)	0 (0)
Event Health Screening (N=17)	0 (0)	3 (18)	0 (0)	0 (0)	0 (0)

^a^To improve clarity and readability of the table, rows and columns with no percentages > 10% have been removed from the table. The full table including all the themes on the columns and rows is provided in Multimedia Appendix 1.

The other most commonly used themes were *recognizing-special days*, *recognizing-employees*, and *sharing-patient success and feel good stories.* Those themes existed in the top 3 themes of 11, 8, and 5 hospitals, respectively. Moreover, in 8 cases, event-related themes were among the commonly used themes by the hospitals. The most frequently used event-related theme was *announcing and reporting events-health information*, which appeared in the top themes of 4 hospitals. *Announcing and reporting events-charity* was the other event-related theme used commonly by 3 of the sampled hospitals.

*Announcing and reporting research activities* was also one of the top 3 themes for 4 hospitals including Brigham and Women’s Hospital, National Jewish Health, Denver-University of Colorado Hospital, Cedars-Sinai Medical Center, and NYU Langone Medical Center. This is consistent with what we expected from hospitals such as Brigham and Women’s Hospital, which is an academic medical center affiliated with Harvard Medical School. Finally, University of Pittsburgh Medical Center included *announcing and reporting-organ donation* information in 9 of its 111 posts, which was unique among the sampled hospitals. Overall, the results showed differences in the ways hospitals used their Facebook pages to communicate with their audience, although some themes were commonly used by several hospitals.

Finally, we examined co-occurrence patterns between themes by calculating the rate at which pairs of themes co-occurred in the sample. Table 5 and Multimedia Appendix 1 present the short and full versions of the co-occurrence analysis results, respectively. Each cell in Table 5 and Multimedia Appendix 1 shows *the percentage of the posts tagged with the theme in the corresponding row that was also tagged with the theme in the corresponding column*. For example, 85 posts in the sample were classified as *announcing and reporting-research activities*. Of those posts, approximately 38% (32/85) were also classified as *sharing-health information*. This means hospitals and clinics frequently use social media to make followers aware of the institutions’ research activities and to inform the audience of the health-related outcomes and issues related to that research. Similarly, 25.4% (45/177) of the posts classified as *recognizing-special days* were at the same time used for *sharing-health information*. It is worth mentioning that, to improve clarity and readability of Table 5, we only included the rows and columns with at least one co-occurrence percentage greater than or equal to 10%. The full table containing the co-occurrence percentages associated with all the ordered pairs of themes is available in Multimedia Appendix 1.

## Discussion

### Principal Findings

The results of this study showed most posts in the sample could be classified as serving 3 purpose groups: *announcing and reporting*, *recognizing*, and *sharing* activities. Within the *announcing and reporting* purpose group, we found the sampled institutions used Facebook to broadcast information relating to *donations* opportunities, upcoming *events*, *research activities*, and *organizational news*. We further found that *recognizing* posts were used to *acknowledge employees* and *special days*, whereas *sharing* posts were used to disseminate *health information* as well as *patient success and feel good stories*.

Over one-quarter of the content posted by the sampled institutions focused on *sharing health-related information*. Accordingly, we believe health care organizations perceive social media as a tool for disseminating health-related information. This is most likely because of information being disseminated very quickly at no cost and remaining persistent on the institution’s Facebook page. This finding is in line with the results of the extant literature (eg, [4,13]) that indicates educating health consumers such as patients and caregivers is among the main reasons health care institutions adopt social media. Furthermore, our findings substantiate CSC’s Global Institute for Emerging Healthcare Practices’ conclusion [5] that health professionals perceive the primary reason for institutional social media use is to promote community wellness and healthy behaviors. Accordingly, health care institutions strive to raise awareness about diseases, medications, nutrition, physical activities, and other health and wellness-related topics to benefit their patients and improve the health of the surrounding community.

The second and third most frequently occurring themes in our sample were *recognizing special days* and *recognizing employees*. These themes were quite interesting, as most previous research has focused on how health care institutions use social media to interact with external stakeholders such as patients, family members, and the surrounding community (eg, [4,13]). The recognizing posts we examined were primarily geared toward stakeholders who were internal to the organization. For example, Nurses Recognition Week fell within our sample timeframe and all of the included institutions made several posts recognizing the nurses in their organizations. In addition, many of the sampled institutions used posts to recognize the contributions and accomplishments of specific individuals and departments within the organization. We believe these acknowledgments increase employees’ satisfaction with the organization and may indirectly promote the quality of health care services provided to the public.

In addition to Nurses Recognition Week, the sampled posts recognized a range of other special days. By acknowledging these days, the organizations may aim to promote health in the society (eg, through promoting National Walking Day), raise awareness about diseases and medical conditions (eg, World No Tobacco Day), or acknowledge the role of people who play major roles in providing health care services to the community (eg, National Doctor’s Day). We believe this finding is an important contribution to the literature as *recognizing employees* and *recognizing special days* were not adequately emphasized in previous studies as main purposes of using social media by health care organizations.

Another difference between our findings and the results of the related studies is although *recruiting employee* and *recruiting volunteers* emerged as 2 themes in our study, only 3 of the 1184 posts the researchers achieved consensus on (<0.3%) were related to recruiting employees or volunteers. This finding is in contrast to the survey of 36 US hospitals and health systems conducted by CSC’s Global Institute for Emerging Healthcare Practices in 2012 [5], which revealed 47% of the respondents indicated they used social media for workforce recruitment. This deviation may imply health care institutions have realized that Facebook is not an effective tool for communicating with health-related job seekers. Instead, those organizations may use professional social media platforms such as LinkedIn for recruitment purposes.

Finally, we found certain themes co-occurred significantly more frequently than others did. For example, 38% of the posts related to *announcing and reporting-research activities* were used to also *share health information* with community members and 12% of the posts *announcing and reporting health screening events* linked the posts back to *organizational research activities*. This may indicate that health care organizations purposely link the content they share to specific initiatives within the organization.

From a practical point of view, our results demonstrated leading hospitals use social media platforms primarily for educating patients on health and wellness topics, announcing and reporting on different types of events, and recognizing employees and special days. These results can be used as a benchmark for the health care institutions that want to establish a social media presence to communicate with the public audience and for the smaller clinics and hospitals that want to further expand and improve their activities on social media websites.

From a theoretical standpoint, our results add to the literature on the uses of social media in health care. Previous studies have reported general intentions for health care institutions’ social media use, and our findings revealed both similarities and differences between the content hospitals share and those intentions. Our results suggest health care organizations’ use of Facebook for recognizing employees, sharing research activities, and announcing organizational news and achievements may be for the purpose of brand recognition. This is in line with previous research in the health care context [5] and other domains such as travel and fashion [6,7] that have found brand recognition to be one of the major reasons organizations use Facebook and other social media platforms. Moreover, to the best of our knowledge, this is among the first studies that empirically and inductively developed a hierarchical thematic web of content of general care health care institutions’ postings on social media websites. This novel research method can further be adopted in different research areas within the medical informatics and health care information systems domains.

### Limitations and Future Research

This study has limitations. For example, we collected data from the Facebook posts made by a sample of 17 hospitals during a 3-month period. Those hospitals and their posts may not represent the activities of all the health care institutions and all social media websites. Future studies can fill this gap by expanding the scope of the sampled hospitals and social media platforms. Collecting post data associated with a longer period will also enable future researchers to perform time series analysis and examine trends of the content generated by clinics and hospitals on their Facebook pages. Another limitation of our study is that the data were collected in 2014. Considering health care institutions may change their social media strategies and activities over time, future studies can collect more recent data to compare and contrast the results with our findings. This will enhance understanding of the evolution of health social media activities by medical institutions.

Building on our results, researchers of future studies can examine the efficacy of different themes by investigating questions such as the following:

1. Does announcing events on Facebook increase attendance?
2. Does acknowledging employees increase their organizational commitment, morale, and satisfaction?
3. Is sharing health information via Facebook an effective approach in raising awareness about diseases and medical issues in the community?

Furthermore, future research can examine user engagement in different types of posts to understand the extent to which each content type can draw users’ attentions and trigger their reactions in terms of liking a post, leaving a comment on it, or sharing it with others on Facebook. Understanding user engagement is important because it can transform one-way, provider-to-consumer information dissemination activities into two-way or many-to-many communication processes. In this way, the “social” aspect of such platforms as Facebook can be realized more meaningfully, adding value to the health care organizations’ activities in virtual environments.

Another limitation of this study is that our sample only included US-based organizations. To enhance the generalizability of results, future studies should include data from a larger sample of international-based health care institutions on different websites such as Twitter, YouTube, LinkedIn, and Yelp during a wider range of time. In addition, health care organization types other than hospitals and clinics could be included in future samples.

Furthermore, we used the thematic network developed in the first phase as a foundation for coding the posts in the second phase of the study. At the same time, we were open to adding new themes that might emerge in the second phase and to removing the themes represented in an infrequent number of posts analyzed. Nevertheless, the coders’ judgments in the coding process during the second phase might still be subject to a level of bias toward the themes identified during the first phase. This limitation could be mitigated in future studies by using automatic, computer-based coding algorithms and tools to objectively validate the hierarchical thematic model developed in this study. Another avenue for future research is to examine the content generation process in terms of who is responsible for posting contents on health care organizations’ Facebook pages, and whether their expertise is in health care (eg, physicians, dentists, and nurse practitioners) or in social media marketing. Such research should investigate how the content provider impacts the type of content shared, the manner in which it is shared, and the resulting level of audience engagement. This can ultimately influence the overall effectiveness of health care organizations’ activities on Facebook and other social media platforms.

### Conclusions

Previous studies in the health care social media domain have predominantly focused on staff members’ perceptions regarding the purpose of social media use for the institution. In this study, we took an alternative approach and focused on thematically analyzing the actual content shared by 17 major health care institutions to understand the different types of content shared by those organizations. Our results provide a robustly validated set of standard content themes as well as information concerning the content themes most frequently shared by the various institutions. Frequency analysis of those themes revealed 2 interesting and unexpected findings. First, a large number of the sampled posts contained information targeted toward stakeholders internal to the organization (nurses, staff, and physicians). This was unexpected as previous studies have focused on how health care institutions use social media to connect with patients, caregivers, and the larger community. Second, very few of the analyzed posts (<0.3%) were geared toward employee recruitment, which previous studies have noted as a key reason staff members believe the institution uses social media. Finally, we identified several co-occurrence patterns within the identified themes, indicating health care institutions often leverage the information in a post to serve more than one objective. We believe these findings can provide a framework that other health care institutions can use to assess their own social media activities to benchmark their activities against a set of nationally recognized institutions.

## Appendix

Multimedia Appendix 1 Theme co-occurrence rates (full version).
