# Supplementary figures and images for: Exploring Hospitals’ Use of Facebook: Thematic Analysis
Source: J Med Internet Res. 2018 May 16;20(5):e190. doi: 10.2196/jmir.9549 (PMC5976865; doi:10.2196/jmir.9549)

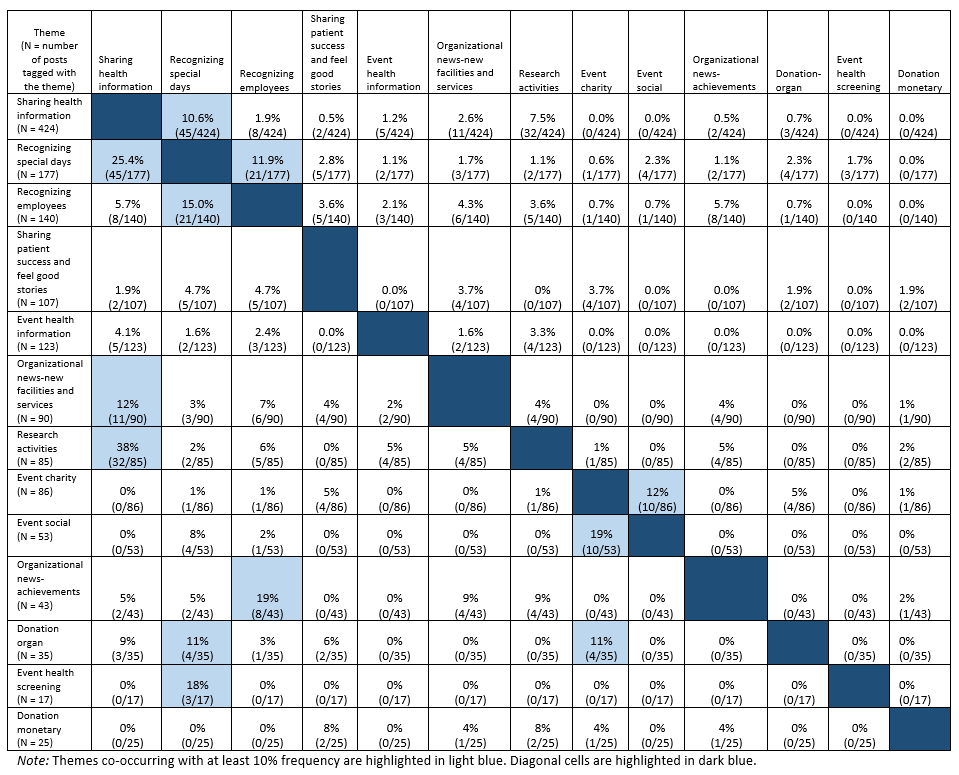

Supplement: Multimedia Appendix 1 [file jmir_v20i5e190_app1.PNG]
